# Anti-Atherogenic Effect of Hydrogen Sulfide by Over-Expression of Cystathionine Gamma-Lyase (CSE) Gene

**DOI:** 10.1371/journal.pone.0113038

**Published:** 2014-11-14

**Authors:** Sau Ha Cheung, Wai Kei Kwok, Ka Fai To, James Yun Wong Lau

**Affiliations:** 1 Department of Surgery, Faculty of Medicine, the Chinese University of Hong Kong, Hong Kong SAR, People's Republic of China; 2 Department of Anatomical and Cellular Pathology, Faculty of Medicine, the Chinese University of Hong Kong, Hong Kong SAR, People's Republic of China; Emory University, United States of America

## Abstract

Hydrogen sulfide (H_2_S) is an important gaseous signaling molecule that functions in physiological and pathological conditions, such as atherosclerosis. H_2_S dilates vessels and therefore has been suggested as an anti-atherogenic molecule. Since cystathionine gamma-lyase (CSE) enzyme is responsible for producing H_2_S in the cardiovascular system, we hypothesized that up-regulation of CSE expression *in vivo* with preservation of H_2_S bioactivity can slow down plaque formation and, can serve as a therapeutic strategy against atherosclerosis. In this study, C57BL/6 wild type mice (WT), ApoE knockout mice (KO) and transgenic ApoE knockout mice overexpressing CSE (Tg/KO) at four weeks of age were weaned. They were then fed with either normal or atherogenic diet for 12 weeks. At week 16, serial plasma lipid levels, body weight, and blood pressure were measured prior to euthanization of the mice and the size of atherosclerotic plaques at their aortic roots was measured. Tg/KO mice showed an increase in endogenous H_2_S production in aortic tissue, reduced atherosclerotic plaque sizes and attenuation in plasma lipid profiles. We also showed an up-regulation in plasma glutathionine peroxidase that could indicate reduced oxidative stress. Furthermore, there was an increase in expression of p-p53 and down regulation of inflammatory nuclear factor-kappa B (NF-κB) in aorta. To conclude, alteration of endogenous H_2_S by CSE gene activation was associated with reduced atherosclerosis in ApoE-deficient mice. Up-regulation of CSE/H_2_S pathway attenuates atherosclerosis and this would be a potential target for therapeutic intervention against its formation.

## Introduction

Atherosclerosis is an inflammatory process that takes place in medium and large sized arteries. It is characterized by plaque formation on the endothelial wall, causing hardening and narrowing of arteries. The process is initiated by accumulation of fatty materials such as cholesterol and triglyceride. Lipid deposition in arteries triggers proliferation of vascular smooth muscle cells (VSMCs) and results in recruitment of circulating inflammatory cells [1]. Macrophages and smooth muscle cells (SMCs) then engulf lipids to form foam cells. With disease progression, VSMCs migrate to intima to form necrotic core which is surrounded by a fibrous cap consisting of VSMCs, collagen and other extracellular matrix (ECM). Plaque rupture occurs by induction of apoptosis of VSMCs and breakdown of collagen and ECM. This causes cerebral or cardiac events [2].

Hydrogen sulfide (H_2_S) is a gaso-transmitter along with nitric oxide (NO) and carbon monoxide (CO) [3]. Similar to nitric oxide, H_2_S is a potent vasodilator [4] and possesses vasoprotective effects, such as reduction of VSMC proliferations [5]. Cystathionine gamma-lyase (CSE) is one of the key enzymes producing endogenous H_2_S and is expressed abundantly in mammalian cardiovascular system [6]. Recently, *in vitro* and *in vivo* studies were carried out to investigate the role of H_2_S in the pathogenesis of atherosclerosis. Deficiency of H_2_S appears to accelerate atherosclerosis. CSE-knockout mice were shown to have lower aortic H_2_S production [7] and were found to develop early fatty streak lesions in the aortic root, elevated plasma levels of cholesterol and low-density lipoprotein cholesterol, hyperhomocysteinemia, increased lesional oxidative stress and adhesion molecule expression, as well as enhanced aortic intimal proliferation after being fed with atherogenic diet [8]. On the contrary, supplementation with H_2_S inhibits atherosclerosis. It was found that H_2_S inhibited ICAM-1 expression in TNFα-induced HUVECs via the NF-κB pathway in ApoE knockout mice [9] and induced superoxide dismutase (SOD) expression, accompanied by a reduced level of reactive oxygen species (ROS) [10]. H_2_S also inhibited macrophage infiltration and reduced lesion size by down-regulation of CX3CR1 and CX3CL1 in macrophages [11], [12]. A recent report also showed that ApoE knockout mice with a H_2_S-releasing drug administration can decrease vascular inflammation and oxidative stress together with improved endothelial function and reduced atherosclerotic plaque formation [13].

Thus far, the relationship between the metabolism of H_2_S and atherosclerosis has been evidenced using CSE knockout mice. CSE gene deletion led to decrease of H_2_S production with accelerated atherosclerosis [8] and this can be ameliorated by treatment with NaHS or exogenous H_2_S donor [8]. However, this is not clear because the proximal regulation of H_2_S at physiological level and toxicological level is ambiguous. Moreover, the *in vivo* level of H_2_S is hard to be manipulated since the release of H_2_S by exogenous H_2_S donor may be time-dependent and/or dose-dependent [14]. CSE inducers or stimulators can be used but little is known.

Therefore, in this study, we hypothesize that endogenous H_2_S can alter the development of atherosclerosis by *in vivo* overexpression of CSE. In addition, *in vivo* overexpression of CSE increases endogenous hydrogen sulfide production. Up-regulation of CSE/H_2_S system may inhibit atherosclerosis.

## Materials and Methods

### Animals and Diets

All animal experiments were approved by the Animal Experimentation Ethics Committee (AEEC) of the Chinese University of Hong Kong (Reference Number: 10/083/MIS) and in accordance with the Animals (Control of Experiments) Ordinance (Cap. 340) licensed from the Hong Kong Government Department of Health. ApoE knockout with C57BL/6 background (KO) mice (The Jackson Laboratory, USA) and transgenic (Tg) mice overexpressing cardiac-specific CSE with C57BL/6 background (from Professor Lefer, Emory University School of Medicine) were crossed to generate CSE transgenic ApoE KO mice, i.e. Tg(CSE)^+/0^ApoE^−/−^ mice (Tg/KO). In the present study, Tg/KO littermates were first characterized and confirmed by genotyping before treatment. Mouse tail of littermates was used for the genotyping using Mouse Genotyping Kit (KAPA Biosystems, Woburn, MA, USA). Genotyping for ApoE locus was done according to the provided by the Jackson Laboratory with the use of the forward primer oIMR0180 5′-CCTAGCCGAGGGAGAGCCG-3′, together with the 2 reverse primers oIMR0181 5′-TGTGACTTGGGAGCTCTGCAGC-3′ and oIMR0182 5′-GCCGCCCCGACTGCATCT-3′ where oIMR0180 and oIMR0181 amplify a 155-bp fragment of the WT locus (ApoE^+/+^), and oIMR0180 and oIMR0182 amplify a 245-bp fragment from the targeted allele (ApoE^−/−^). The MHC-CSE transgene was confirmed by PCR reaction using forward primer 5′-TGTTGGATTTCCAGAGCGGCTGTA-3′ and reverse primer 5′-TCTTAGCAAACCTCAGGCACCCTT-3′ to amplify a 420-bp transgene (Tg(CSE)^+/0^).

C57BL/6 mice (WT), ApoE^−/−^ mice (KO) and Tg(CSE)^+/0^ApoE^−/−^ mice (Tg/KO) were housed in a temperature-controlled room with free access to food and water and maintained on a 12-h light/dark cycle. At 4 weeks of age, mice were weaned and fed either normal diet (Harlan Laboratories, Madison, WI, USA) or atherogenic diet (MP Biomedicals, Aurora, OH, USA) for 3 months. Body weight of each mouse was recorded every week and the weight gain was calculated at the end of the study. Daily food intake to both groups of mice was measured for a week. Blood pressure was also measured at the end of the study using a non-invasive computerized tail cuff system (Kent Scientific Corporation, Litchfield, CT, USA). 6 readings were taken and an average of readings was used. The mice were euthanized at the age of 16 weeks for isolation of tissue for examinations. They were first anaesthetized by injection of ketamine (50 mg/kg im), followed by immediate termination. All efforts were made to minimize suffering.

### CSE Quantitation by RT-PCR

Total RNA from aorta and heart tissue were separately homogenized and extracted with Trizol reagent (Invitrogen, Burlington, Ontario, Canada) according to the manufacturer's protocol. The concentration of total RNA was determined by Nanodrop ND-1000 Spectrophotometer (Thermo Fisher Scientific, DE, USA). cDNA synthesis was performed according to the protocol provided with M-MLV reverse transcriptase (Promega Corporation, WI, USA) using 200 units of enzyme, 0.5 µg of random primers (Invitrogen, USA) as primers, and 1 µg of RNA. Real time RT-PCR was carried out using aorta cDNA as template and analyzed in an ABI 7500 Fast Real-Time PCR Systems (Applied Biosystems, Foster City, CA, USA) using fluorescent TaqMan methodology. Real time quantitative RT-PCR was performed using ready-to-use primer and probe sets pre-developed by Applied Biosystems (TaqMan Gene Expression Assays) to quantify mouse CSE and mouse Glyceraldehyde-3-phosphate dehydrogenase (GAPDH). mRNA Ct values for these genes were analyzed on SDS v1.4 Software (Applied Biosystems) and normalized to the house-keeping gene GAPDH. Expression of CSE gene was then validated by running the PCR products on a 2% (w/v) agarose gel.

### Protein Expression in Aorta by Western Blotting

Mice aorta was lysed at 4°C in radio-immunoprecipitation assay (RIPA) lysis buffer. The lysate was clarified by centrifugation at 14,000 g for 15 min at 4°C. Protein concentration in the supernatant was determined by BCA protein assay kit (Pierce Biotechnology, IL, USA). Protein samples (50 µg) were separated by 10% SDS-PAGE and then transferred into nitrocellulose membranes (Bio-rad Laboratory, Hercules CA, USA). The membrane was blocked with 3% bovine serum albumin (BSA) in Tris buffered saline (TBS)-Tween buffer and then probed overnight at 4°C with anti-CSE (1∶1000; Abnova, Taipei City, Taiwan), anti-phosphorylated p53 (1∶1000; Cell Signaling Technology, Danvers, MA, USA), anti-IKK (1∶1000, Cell Signaling Technology, Danvers, MA, USA), anti-IκBα (1∶1000, Cell Signaling Technology, Danvers, MA, USA), anti-NF-κB p65 (1∶1000, Cell Signaling Technology, Danvers, MA, USA) and anti-GAPDH (1∶2000; Santa Cruz Biotechnology, CA, USA). It was followed by secondary antibody for 1 h with a 1∶2000 dilution of horseradish peroxidase (HRP)-conjugated, goat anti-mouse IgG (Cell Signaling Technology, Danvers, MA, USA) or goat anti-rabbit IgG (Cell Signaling Technology, Danvers, MA, USA). After washing, the membrane was developed with ECL kit (Amersham Pharmacia Biotechnology, Buckinghamshire, UK) and exposed to X-ray films (Amersham Pharmacia Biotechnology, Buckinghamshire, UK). Intensity of bands was quantified using the public domain software, ImageJ software.

### Determination of Plasma Lipids and Adiponectin

Mice were fasted for 16 to 20 h prior to sacrifice. Blood samples were drawn by cardiac puncture using 25G needle mounted on a 1 ml syringe and were collected in EDTA tube. Plasma was separated by centrifugation at 2000 g for 10 min and stored at −80°C. Plasma lipid concentrations including total cholesterol and low-density lipoprotein (LDL) cholesterol were measured by quantitative colorimetric techniques according to the manufacturer's procedures (BioAssay Systems, CA, USA). Plasmatic triglyceride level was determined by another commercial assay kit (Wako Pure Chemical Industries Ltd., Osaka, Japan). We measured adiponectin in plasma using an enzyme-linked immunoassay (SPI-BIO Co., Montigny-le-Bretonneux, France).

### Hydrogen Sulfide Production in Heart Tissues

Synthesis of hydrogen sulfide (H_2_S) in heart tissue homogenates was measured as described by Wallace group [15] with some modifications. Mice heart was quickly isolated and snap-frozen. Heart tissue was homogenized in tissue protein extraction reagent (Thermo Pierce, Rockford, IL, USA). The homogenate (0.55 ml) and buffer (0.45 ml) were then cooled on ice for 10 min before l-cysteine (50 µmol/L) and pyridoxal 5′-phosphate (10 mmol/L) were added. The final volume was 1 ml. A smaller 0.5-ml eppendorf containing a piece of filter paper (0.5×2.5 cm) soaked with zinc acetate (1%; 0.5 ml) was put inside the larger vial. The vials were then flushed with nitrogen gas for 20 sec and capped with an airtight serum cap. The vials were then transferred to a 37°C shaking bath for 5 h and stopped by injection of trichloroacetic acid (TCA; 50%; 0.5 ml) into the reaction mixture through the serum cap. The mixture was left to stand for another 60 min to allow for the trapping of evolved H_2_S by the Zinc acetate. The serum cap was then removed and N, N-dimethyl-p-phenylenediamine sulfate (20 mmol/L; 50 µl) in 7.2 mol/L HCl and FeCl_3_ (30 mmol/L; 50 µl) in 1.2 mol/L HCl were added to the inner tube. After 20 min, absorbance at 670 nm was measured with a microplate reader (Bio-Tek Instrument INC., Rockville, MD, USA). The calibration curve of absorbance versus H_2_S concentration was obtained by using NaHS solution of varying concentrations (0 to 320 µm). Results were then corrected for the protein content of the tissue sample and expressed as µmoles H_2_S generated/mg protein/h.

### Quantitation of Atherosclerotic Lesion

To quantify the atherosclerotic lesions in the aortic root, upper part of heart was cut in the ascending aorta and the proximal sample containing the aortic sinus was embedded in Tissue Tek OCT (Optimal Cutting Temperature) compound (Miles Scientific, Naperville, IL, USA) and then stored at −80°C. Serial cryostat sections (6 µm) were prepared on a cryostat (Leica Microsystems, Buffalo, NY, USA). In brief, atherosclerotic lesions in the aortic root were examined at 6 locations and each separated by 90 µm, 9 serial sections were prepared from each location. These sections were stained with oil red O and counterstained with Mayer's hematoxylin. Whole slide images were produced with Leica SCN400 slide scanner (Leica Biosystems, Buffalo Grove, IL, USA), managed with the image server, Digital Image Hub (Leica Biosystems). In each case, average value for 3 serial sections at each of 6 locations of each animal was used for analysis. The lipid composition of the lesion was determined by calculating the percent of the oil red O positive area to the area of aorta root using the public domain software, ImageJ software. The area of aorta root was manually selected and the positive stained area within the aorta root was measured by adjusting the threshold value. The remaining sections were used for immunohistochemical analysis as described below.

### Immunohistochemistry of CSE in Aortic Roots

Air-dried cryostat sections (6 µm thickness) of aortic root were fixed with the pre-cooled acetone for 10 min at room temperature and rinsed with PBS three times. Endogenous peroxidase activity was inactivated by treatment in 3% H_2_O_2_ for 10 min, followed by a wash in PBS. Non-specific staining was blocked by incubation with 2% goat serum in PBS for 30 min and washed in PBS. Slides were then incubated with primary antibody, anti- CSE (Santa Cruz Biotechnology, CA, USA) in a 1∶50 dilution at 4°C overnight, followed by secondary antibody in a 1∶200 dilution of HRP-conjugated, anti-goat IgG (Cell Signaling Technology, Danvers, MA, USA) for 1 h at room temperature. Immunohistochemical staining was developed by exposure to 3,3′-diaminobenzidine (DAB) and counterstaining was performed with hematoxylin. Whole slide images were produced as described above.

### Oxidative Stress Measurement

Commercially available glutathionine peroxidase (GPx) assay kit (Cayman Chemical Company, MI, USA) was used to measure plasma GPx activity according to the manufacturer's instruction. Glutathione and total glutathione were measured using glutathione colorimetric assay (BioVision Incorporated, CA, USA).

### Statistical Analysis

All data are presented as the mean ± SEM. Non-parametric t-test was used to compare variables between two groups. For the correlation study, for example CSE expression versus total cholesterol and H_2_S production rate versus NF-κB expression, was performed by simple linear regression analysis. For all tests, *P*<0.05 was considered statistically significant. A complete list of the underlying data set was shown in Table S1.

## Results

### Role of CSE Expression in Hydrogen Sulfide Production Rate in Heart

**Figure 1:**
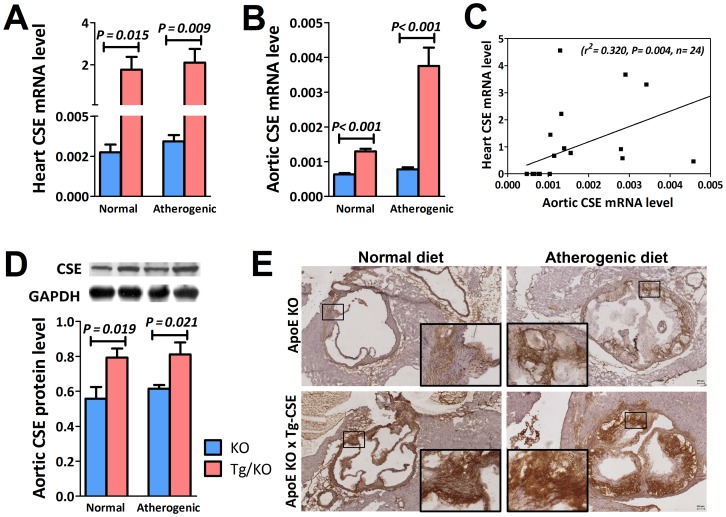
CSE expression in heart and aorta. CSE mRNA levels of (A) heart tissue (n = 6) and (B) aorta (n = 6) in Tg/KO mice were increased when compared to KO mice. (C) The relationship between the heart CSE mRNA level and aortic mRNA level was significantly positive correlated. (D) Besides, protein level (n = 6) was significantly increased in aorta homogenates of Tg/KO mice on either normal or atherogenic diet. (E) Representative immunohistochemical staining also indicated higher CSE expression in aorta root in Tg/KO mice as compared to the KO mice in both diets (Magnification 4×; Insert magnification 20×).

**Figure 2:**
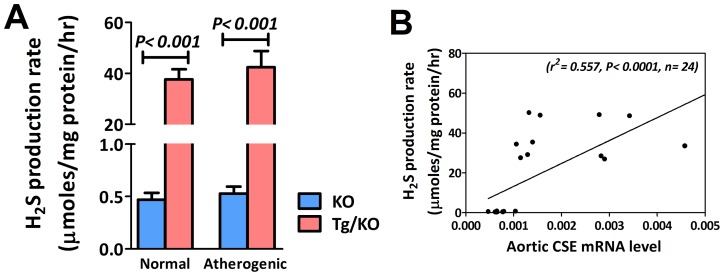
Direct relationship between aortic CSE expression and H_2_S production. (A) With overexpression of CSE gene in Tg/KO mice, H_2_S production in heart was considerably up-regulated (n = 6). (B) Direct relationship between aorta CSE expression and heart H_2_S generation was also revealed, showing significant positive correlation between them.

To investigate the effect of aortic CSE gene activation on endogenous H_2_S production in mice heart, CSE expression levels and H_2_S production were assessed in CSE transgenic ApoE-KO (Tg/KO) mice and ApoE-KO (KO) mice fed with both normal and atherogenic diets for 12 weeks. As illustrated in Figure 1, CSE gene was detected in heart tissue (Figure 1A) as well as in aorta (Figure 1B). The association of CSE mRNA expression between the heart tissue and aorta was found positive correlated (Figure 1C). This indicates that overexpression of CSE gene can cause an increase of CSE expression in aorta. We then examined CSE protein level in aorta. The protein by western blotting was around 1.3 fold increased (Figure 1D) in Tg/KO mice as compared to the corresponding KO mice. Using immunohistochemistry, expression of CSE was detected in the aorta root. CSE was abundantly expressed in aorta of Tg/KO mice in both diets (Figure 1E). To elucidate the role of CSE in H_2_S production, H_2_S generation from heart was also markedly elevated in Tg/KO mice fed with either normal or atherogenic diets (Figure 2A). The relationship between aorta CSE expression and H_2_S production rate from heart also illustrated. There was a significant positive correlation between the aorta CSE expression and the H_2_S production in heart (Figure 2B). This indicates that CSE gene is the vital enzyme for the H_2_S production in cardiovascular system.

### Body weight and blood pressure

**Figure 3:**
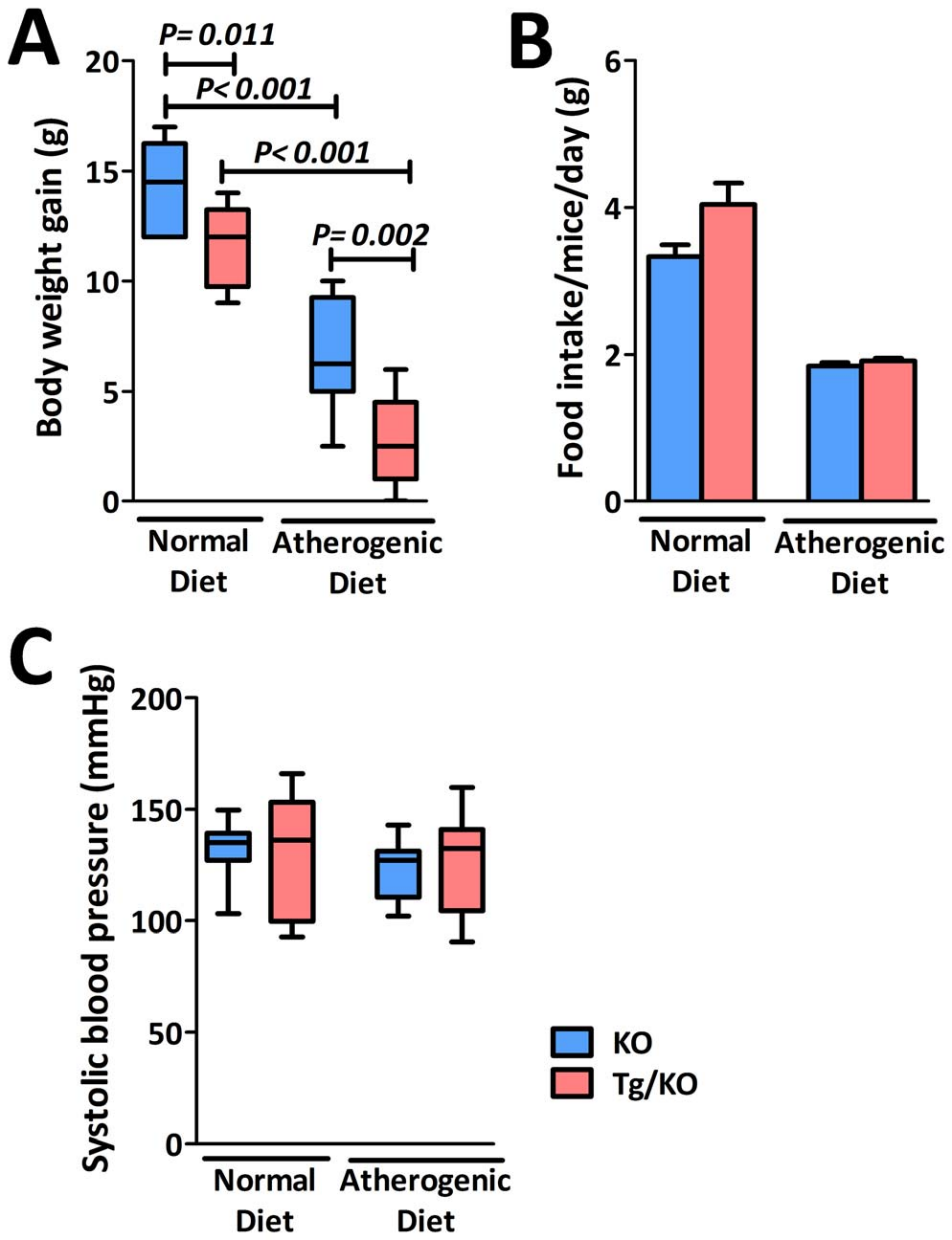
Body weight and systolic blood pressure in CSE transgenic ApoE-KO (Tg/KO) mice. (A) Body weight gain changes during the feeding periods with different diets. Both KO and Tg/KO mice (n = 10) showed increase of body weights with same diet but the weight gain was significantly different. Mice fed with normal diet had higher weight gain than that with atherogenic diet. Moreover, it also showed that Tg/KO mice had fewer weight gains in both normal and atherogenic diets as compared to KO mice. (B) Daily food intake was similar in both KO and Tg/KO (n = 6). (C) Both KO and Tg/KO mice (n = 10) did not have significantly change with the systolic blood pressure, indicating that Tg/KO mice did not cause hypotension after insertion of CSE gene.

**Figure 4:**
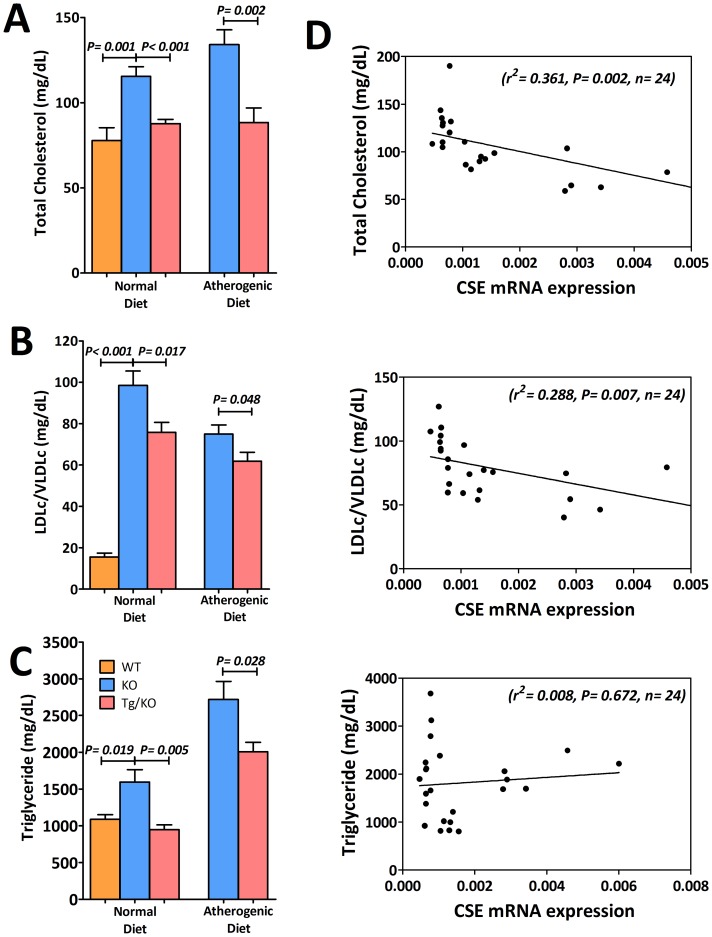
Anti-atherogenic effect of CSE gene activation with higher H_2_S production. (A) Plasma total cholesterol (n = 8 to 10), (B) LDL/VLDL cholesterol (n = 8 to 10) and (C) triglyceride level (n = 6) were significantly reduced in Tg/KO mice after feeding with 12 weeks of normal diet or atherogenic diet when comparing to KO mice. (D) Relationship between aortic CSE mRNA expression level and levels of plasma lipids. There was a significant negative relationship between CSE mRNA expression and levels of total cholesterol and LDL/VLDL cholesterol. However, its relationship with triglyceride was not statistically significant.

During the 12 weeks of diet treatment, both KO and Tg/KO mice increased in their body weight. But their weight gain was significantly reduced once the diets switched from normal to atherogenic at 4 weeks of age (Figure 3A). When comparing Tg/KO mice with KO mice with the same diet, the average weight gain by Tg/KO mice was significant less by about 3 grams (Figure 3A). To demonstrate whether caloric restriction Tg/KO mice could account for cardioprotective effect, food consumption by KO and Tg/KO mice was measured (Table S2). Both KO and Tg/KO mice consumed less in atherogenic diet (Figure 3B). Moreover, the two groups did not differ in the amount of food intake (Figure 3B). Since KO mice shows hypertension in comparison with WT mice groups, it was suspected that overexpression of CSE may lower blood pressure or even cause hypotension in Tg/KO mice. As shown in Figure 3C, CSE gene activation did not significantly change the blood pressure of Tg/KO mice fed with either normal or atherogenic diets. CSE does not appear to affect blood pressure in these mice. Instead, blood pressure seemed to be affected by the level of cholesterol. As shown in Figure 4A, although the total cholesterol of Tg/KO mice was reduced when compared to the KO mice, it was not as low as that of the WT mice.

### Anti-atherogenic effects of CSE/H_2_S

**Figure 5:**
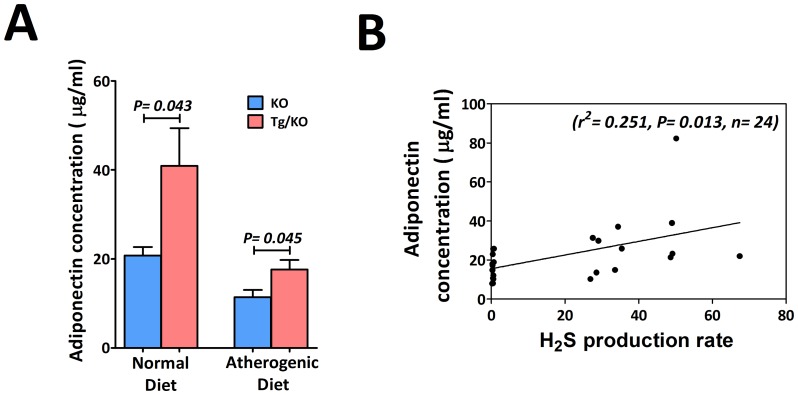
Increased plasma level of adipinectin in CSE transgenic ApoE-KO (Tg/KO) mice. (A) Plasma adiponectin level (n = 6) was higher in Tg/KO mice when compared to the KO mice in both diets. (B) Relationship between plasma level of adiponectin and H_2_S production rate was positively correlated.

**Figure 6:**
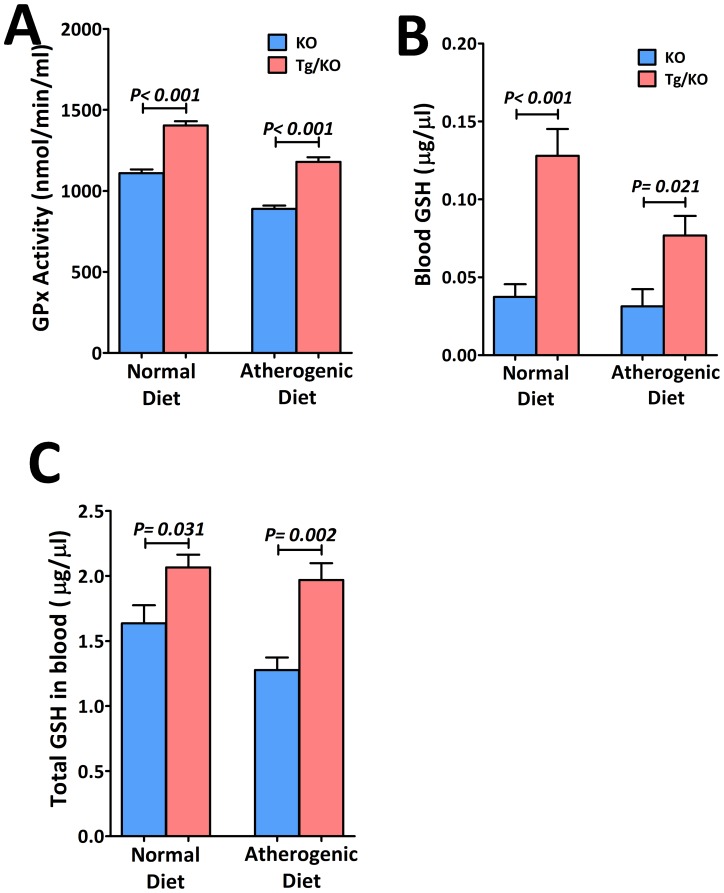
Reduced level of oxidative stress by activation of CSE gene. Improved plasmid lipid levels in Tg/KO mice exhibits reduced level of oxidative stress as indication by higher level of (A) glutathionine peroxidase (GPx) in plasma (n = 6), (B) blood glutathionine (GSH) (n = 6) and (C) total glutathionine (both GSH and GSSG) (n = 6).

With the increase of endogenous H_2_S, plasma total cholesterol (Figure 4A), LDL/VLDL cholesterol (Figure 4B) and triglyceride (Figure 4C) levels were lowered by 24.1%, 23.1% and 40.7% in respectively in mice fed with normal diet. In mice fed with atherogenic diet, the corresponding decrease were 34.1%, 17.4% and 26.3% respectively. Figure 4D showed that there was a significant negative relationship between aortic CSE expression level and both total cholesterol and LDL/VLDL cholesterol. However, its relationship with triglyceride was not statistically significant. Tg/KO mice were found to have higher plasma adiponectin level as compared to KO mice (Figure 5A). The plasma adiponectin level was positively correlated to the H_2_S production (Figure 5B). On the other hand, it was expected that the oxidative stress would be reduced due to the reduced level of LDL/VLDL cholesterol. Therefore, plasma glutathione peroxidase (GPx) activity was determined. In association with attenuation of lipids, markedly elevated plasma GPx activity (Figure 6A), glutathione (GSH) (Figure 6B) and total glutathione/oxidized glutathione (GSH and GSSG) (Figure 6C) were observed in Tg/KO mice fed with both diets, demonstrating that cells are protected from oxidative damages. Overexpression of CSE can help improving plasma lipids and reducing oxidative stress.

### CSE overexpression decelerated atherosclerotic development

**Figure 7:**
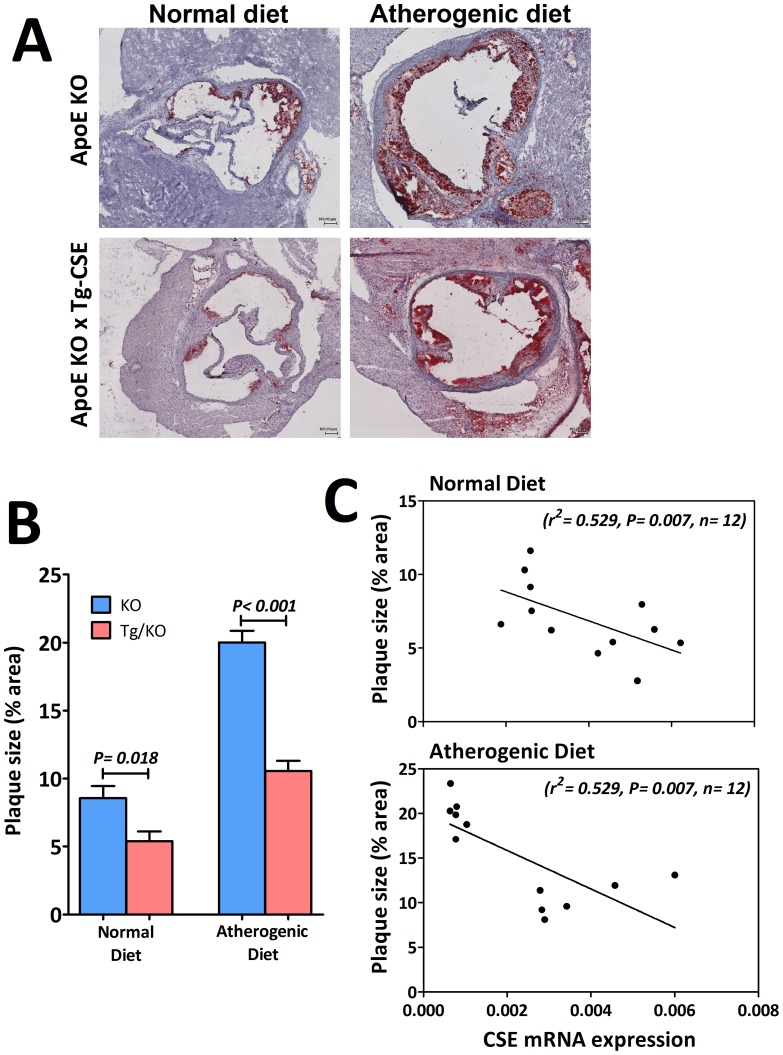
Overexpression of CSE inhibits ApoE deficient mice from atherosclerosis. (A) Atherosclerotic lesion damages in Tg/KO mice improved in both diets when compared to KO mice (Magnification 4×). (B) Quantitative comparisons of the mean percentage of plaque areas in aortic root between KO and Tg/KO mice were calculated using ImageJ software (n = 6). (C) There was a significant negative correlation between aortic CSE mRNA expression and lesion size from aortic root in mice who received both normal diet and atherogenic diet.

To gain further insight on the functional role of CSE on the atherosclerosis development, fatty lipids in aorta root were stained with oil red O to show the extent of lesion area in KO mice and Tg/KO mice. As demonstrated in Figure 7A, KO mice fed atherogenic diet for 12 weeks showed significantly more aortic oil red O staining as compared to the KO mice fed with normal diet. Interestingly, Tg/KO mice fed with either normal or atherogenic diet showed diminished of aortic lipid deposition by approximate 50% as comparing with KO mice fed with the same chow (Figure 7B). To show whether overexpression of CSE could reduce lesion area in aorta, correlation between the CSE mRNA expression in aorta and aortic lesion area was performed in respect to different diets. It was shown that activation of CSE gene can significantly attenuate plaque accumulation in aorta in both normal and atherogenic diets (Figure 7C).

### Anti-inflammatory response of CSE/H_2_S

**Figure 8:**
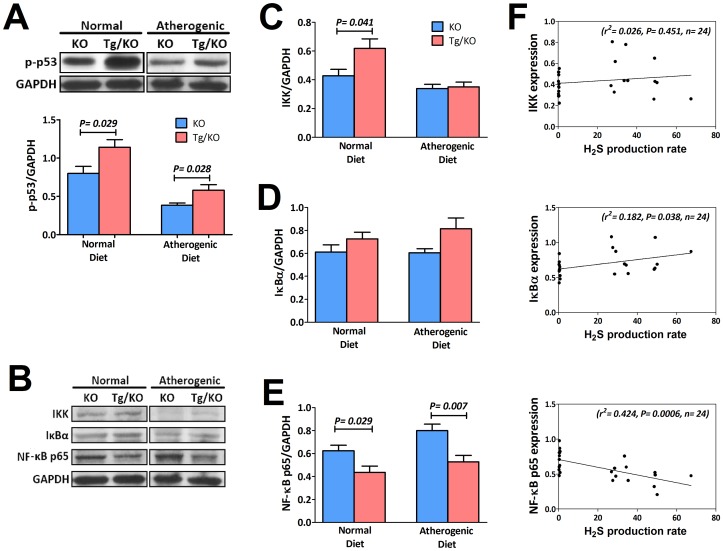
Quantitative analysis of protein expression levels in the aorta of KO and Tg/KO mice. (A) Representative immunoblot showed that p-p53 increased in Tg/KO mice when compared to those in KO mice (n = 6). (B) Immunoblot showed the expression levels of NF-κB pathway including (C) IKK, (D) IκBα and (E) NF-κB p65 (n = 6). Correlation of each protein expression in (B) with H_2_S production was shown in (F).

As an anti-inflammatory molecule of CSE/H_2_S, tumor suppressor gene p53 becomes activated to prevent atherosclerosis by inhibition of cell proliferation and promotion of apoptosis in vascular smooth muscle cells. We quantitatively analyzed the expression of aortic p-p53 as shown in Figure 8A. p-p53 was show to be highly expressed in aorta of Tg/KO mice fed with both normal and atherogenic diets. We then examined the involvement of nuclear factor-kappa B (NF-κB) in both KO and Tg/KO mice as markers for vascular inflammation. As shown in immuneblot (Figure 8B), H_2_S increased the content of IκBα by activation of IκB kinase (IKK) in Tg/KO mice with both diets. Although expression of IKK (Figure 8C) was not significantly increased in Tg/KO mice, the elevated level of IκBα (Figure 8D) represented a suppression of IκBα degradation and subsequently resulted in significant inhibition of NF-κB (Figure 8E). Between, expression of IκBα and NF-κB were found correlated with the H_2_S production (Figure 8F). Activated p-p53 and reduced NF-κB implied anti-inflammatory response induced by CSE/H_2_S.

## Discussion

CSE/H_2_S pathway has been implicated to play an important role in physiologic and pathophysiologic processes of various cardiovascular diseases, including atherosclerosis [10], [16]. H_2_S has been shown to protect cardiovascular tissue by acting as an anti-inflammatory molecule [17], [18]. H_2_S deficiency by CSE knockout leads to decrease in endogenous H_2_S level and development of advanced atherosclerotic lesion [10]. CSE/H_2_S overexpression *in vitro* has been shown to stimulate VSMC apoptosis [19] and inhibit inflammatory response [12]. In this study, we produced CSE transgenic ApoE-KO (Tg/KO) mice to examine the anti-atherogenic effect of H_2_S as compared to ApoE-KO (KO) mice, a mice model for atherosclerosis. Our study shows that *in vivo* CSE/H_2_S overexpression protects ApoE-KO mice from atherosclerosis.

In an earlier study, Yang et al. showed that CSE over-expression of CSE in aortic smooth muscle cells can increase hydrogen sulfide production [19]. We have shown that *in vivo* CSE/H_2_S overexpression in ApoE-KO mice can significantly increase CSE mRNA and protein levels in aorta as well as endogenous H_2_S production in heart. We also found that the elevated level of H_2_S in the heart tissue of Tg/KO mice was associated with the reduced plasma lipids. In healthy subjects, Jain SK et al. measured fasting blood H_2_S and cholesterol levels. They found a positive correlation between blood H_2_S and HDL-cholesterol [20]. In the same study, blood H_2_S positively correlated with adiponectin. In patients with type 2 diabetes mellitus and coronary artery disease, adiponectin level was found to correlate with HDL cholesterol [21]. The effect of H_2_S on cholesterol is thought to be mediated through adiponectin. Our results suggest that H_2_S regulates plasma lipid metabolism through a high plasma adiponectin level. This may lead to reduced oxidative stress to arterial walls and therefore protects against atherosclerosis. Oxidized LDL is important not only for the formation of fatty streaks but also, along with other physical and/or humoral mediators, for damage to the endothelium. The damaged endothelium allows for continued transport of inflammatory cells and mediators into the vessel wall. These processes generate ROS and increase oxidative stress [22].

With increased level of H_2_S in Tg/KO mice, H_2_S reacts with ROS and induces increased production of anti-oxidants such as glutathione (GSH) and glutathione peroxidase (GPx) [23]. GSH and GPx are intracellular antioxidants. Increased GPx protects against oxidative stress [24]. In our study, we have shown an elevated level of GPx as well as increased level of GSH and GSSG in Tg/KO mice, indicating reduced oxidative stress by CSE/H_2_S. Our findings are consistent with those from another report that H_2_S exerts an anti-atherogenic effect in association with elevated plasma GPx [16]. In this study, smaller body weight gain was observed in Tg/KO mice and unfortunately, we could not explain by the dietary experiment. However, interestingly, GSH metabolism has been linked to energy expenditure, insulin sensitivity and body weight regulation [25]. In an earlier placebo-controlled study in healthy subjects, Lands et al. supplemented a whey-based cysteine donor to augment intracellular GSH [26]. They found improved muscular performances in those who received cysteine, which is substrate required for biosynthesis of GSH. In rats, Haraguchi et al. found that whey protein supplements could lead to reduced body and muscle weight gain [27]. In addition, Yang et al. showed a paradoxical relationship between adiponectin and body weight [28]. In another study, adiponectin production in plasma was found to be decreased in obese patients [29]. Adiponectin may therefore inhibit weight gain. Taken together, high plasma adiponectin level and GSH level, which were associated with high H_2_S production rate in this study, could at least in part explain the reduced body weight gain in Tg/KO mice when compared to the KO mice.

Tumor suppressor gene p53 plays an important role in protection against atherosclerosis and restenosis [30]. Its expression was found negatively correlated with cell proliferation in human atherosclerosis [31], but directly associated with increased apoptosis [32], [33]. With regard to H_2_S, many studies have been suggested that H_2_S up-regulated p53 expression to activate cell apoptosis and inhibit cell growth [34], [35], [36]. Baskar and colleagues demonstrated that a H_2_S-releasing donor (S-diclofenac) inhibited rat VSMC proliferation associated with induced p53, p53AIP1 and the transcriptional factors of p53 such as p21 and Bax proteins [37]. In gastric cancer cells, a new H_2_S-releasing donor (SPRC) has been found to increase the expression of CSE and also induced a pro-apoptotic effect in cancer tissues with elevated expressions of p53 and Bax in tumors and cells [38]. Therefore, it is believed that altered metabolism of CSE/H_2_S pathway plays role in cell proliferation of VSMCs and the pro-apoptosis effect of CSE/H_2_S on VSMCs may have important implications in the vascular remodeling progress and the development of atherosclerosis [39].

We showed an anti-inflammatory effect of CSE/H_2_S overexpression in our Tg/KO mice. Oxidative stress stimulates atherosclerosis by increasing production and translocation of NF-κB [9] which is activated by a multitude of stimuli such as cytokines, oxidized lipids, lipopolysaccharide (LPS). The reduced oxidative stress by CSE/H_2_S overexpression may inhibit inflammation via NF-κB signaling pathway. Activation of NF-κB occurs in an IκB kinase complex (IKK)-independent manner and through the tyrosine phosphorylation of IκBα and degradation of IκBα, followed by nuclear translocation of the activated NF-κB dimer to initiate gene transcription [40]. Dysregulation of the NF-κB system is likely to play an important role in inflammatory diseases [41] and also be involved in inflammatory-proliferative process of atherogenesis [42], [43]. With increase of oxidative stress in atherosclerosis, ApoE-KO mice showed accumulation of plaque area with increased IκBα degradation and NF-κB expression. Conversely, Tg/KO mice showed inhibited plaque formation and NF-κB expression. There are evidences to show that exogenous H_2_S can inhibit NF-κB activation in cells stimulated with TNFα [44] and lipopolysachharides (LPS) [17], [18]. Furthermore, overexpression of CSE in macrophage exhibited reduced inflammatory pathway by suppression of IκBα degradation and NF-κB p65 nuclear translocation [13], [24]. However, there is no direct evidence showing anti-atherogenic effect of H_2_S on suppression of NF-κB except a recent study showed that CSE/H_2_S deficiency was associated with increased NF-κB activation [9]. CSE/H_2_S overexpression in our study appears to support an anti-inflammatory effect in the protection against atherosclerosis.

In conclusion, we have shown for the first time that H_2_S protects against atherosclerosis by CSE gene activation. Endogenous H_2_S prevents the progression of atherosclerosis by reducing plasma lipid accumulation, inhibiting oxidative stress and plaque formation and suppressing inflammation in vascular tissues via NF-κB pathway. Therefore, our study provides direct evidence that H_2_S is an important mediator in atherosclerosis formation. The CSE/H_2_S pathway can be targeted as one of the new therapeutic strategies in prevention of atherosclerosis.

## Supporting Information

Table S1 **A complete list of data set in this study.**
(XLS)

Table S2 **Daily food intake per mice.**
(XLS)
